# Sleep apnea: Do not forget to inspect the throat!

**DOI:** 10.1002/ccr3.1927

**Published:** 2018-11-22

**Authors:** Christian Alain Pirovino, Roland Giger, Basile Nicolas Landis

**Affiliations:** ^1^ Department of Otorhinolaryngology ‐ Head and Neck Surgery Bern University Hospital, Inselspital Bern Switzerland; ^2^ Rhinology‐Olfactology Unit, Department of Otorhinolaryngology ‐ Head and Neck Surgery University Hospital of Geneva Geneva Switzerland

**Keywords:** lipoma, obstructive sleep apnea syndrome, parapharyngeal, tiredness

## Abstract

Obstructive sleep apnea syndrome (OSAS) is a widespread and underdiagnosed disease. Causes are mostly related to obesity and anatomy with oro‐pharyngeal narrowing. Parapharyngeal tumors are rare but can easily be treated. Careful oro‐pharyngeal examination in OSAS patient is cheap, easy to perform by non‐ENT specialists, quick, and avoids inadequate treatment.

## 1 INTRODUCTION

Obstructive sleep apnea syndrome (OSAS) is a widespread disease. Unfortunately, the clinical presentation is not always homogeneous with some patients complaining of very vague symptoms like morning headache and daytime tiredness. Especially in patients living alone, the main OSAS symptoms like snoring and apnea remain unnoticed. Obstructive sleep apnea syndrome has repeatedly been reported to be underdiagnosed within the general population.^1^ Untreated OSAS is a risk factor for stroke and it is associated with decreased life expectancy. The financial burden for the society due to undiagnosed and untreated OSAS is considerable. In children, the overwhelming majorities of cases are obstructive and relate to adenotonsillar hypertrophy, whereas adults show a more multi‐factorial etiology of OSAS. Besides maxillo‐mandibular hypotrophies, decreased muscle tone, and narrow naso‐oro‐hypopharyngeal anatomy related to obesity, rare causes such as parapharyngeal tumors, most them benign, may also lead to OSAS.^2^ The present case underlines the importance of a meticulous ENT examination in patients with suspected OSAS.

## 2 CASE REPORT, TREATMENT, OUTCOME, AND FOLLOW‐UP

A 54‐year old moderately obese (BMI = 30.9 kg/m^2^) man with a past medical history of tonsillectomy due to recurrent tonsillitis was referred to our clinic by an external ENT physician for resection of a suspected parapharyngeal lipoma. The patient had suffered from recent onset of an increasing foreign body feeling associated to daytime sleepiness and impaired concentration at workplace for over a year. The patient's general practitioner had suspected a sleep disorder and sent him to a pneumologist a year previous to our referral. The sleep exam revealed an apnea‐hypopnea index (AHI) of 42 per hour compatible with moderate to severe OSAS. Without any further examination, the patient received a continued positive airway pressure (CPAP) for the next 12 months. Despite initial improvement of the sleep architecture and day symptoms, the patient's complaints re‐occurred and foreign body feeling increased.

**Figure 1:**
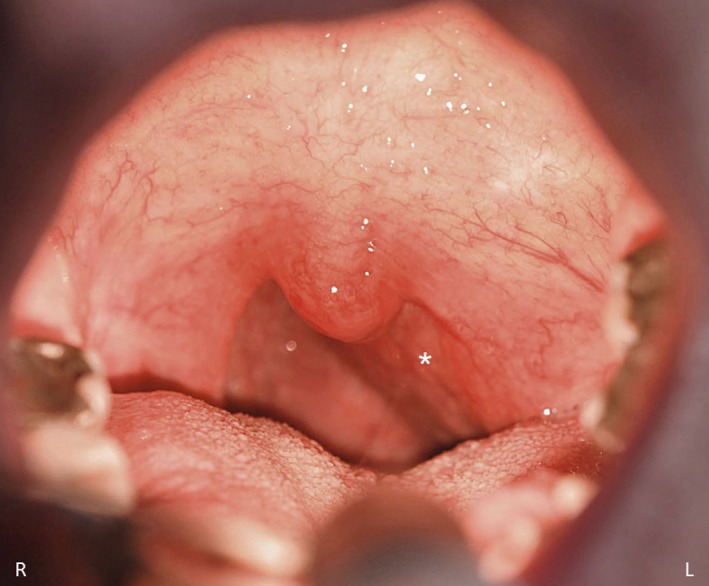
Transoral examination of the oropharynx showing an extensive bulging of the lateral pharyngeal wall (white star)

**Figure 2:**
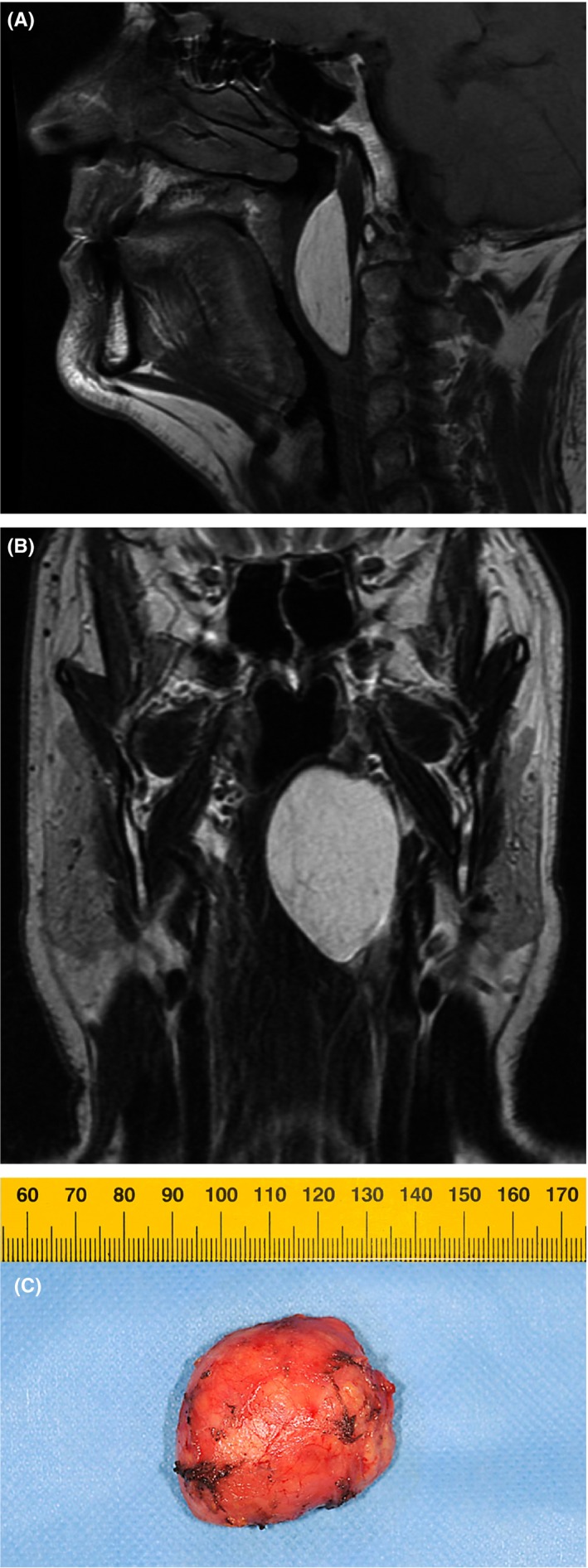
Sagittal (A) and coronal (B) MRI sections (T1‐sequences) of the upper airways. The images show a homogenous parapharyngeal mass adjacent to the masticator space laterally, extending to the retropharyngeal and carotid space dorsally. The mass is narrowing the upper airways at the level of the oropharynx. There are no signs of malignancy. (C) Resected specimen of the well‐defined and encapsulated (histologically confirmed) lipoma

Due to this symptom recurrence further investigations were needed, and he was sent to an external ENT colleague who discovered a bulging of the left tonsillar fossa and lateral pharyngeal wall. Examination of the oropharynx revealed a firm submucosal mass of the parapharyngeal space extending from the lower one‐third of the nasopharynx to the lower aspect of the tonsillar fossa (Figure 1). Magnetic resonance imaging (MRI) showed a 6 × 4 × 2 cm homogenous parapharyngeal mass which appeared hyperintense on T1‐weighted sequences and hypo‐intense on T2‐weighted sequences (Figure 2A,B). The mass extended beyond the midline to the right side and caused stenosis of the upper airway with no neck lymphadenopathies. The radiological pictures highly suggested the presence of a large lipoma of the parapharynx. A complete surgical excision of the mass was performed (Figure 2C) using a transoral approach. Histolopathologic examination confirmed the suspected diagnosis of a lipoma. The post‐operative course was uneventful, and the patient was discharged home on the second post‐operative day. One month following surgery, the patient reported complete relief of all day symptoms. Follow‐up sleep exam 4 months postoperatively showed a decrease of AH index from initially 42 to 11 per hour (without CPAP) and a decrease of the Epworth Sleepiness Scale from 6 to 3 out of 24 points.

## 3 DISCUSSION AND CONCLUSION

The present case impressively illustrates how important careful examination of the upper airway anatomy is when OSAS is diagnosed. Although most OSAS cases in adults have multiple etiologies^3^ such as excess weight, eating, and drinking habits, decreased nocturnal muscle tone or central nervous system causes, it should not be forgotten that pure obstruction of the upper airways by any obstacle may also lead to sleep apnea.^1^ While this is almost exclusively the case in children with adenotonsillar hypertrophy, this is much rarer in adults. Consequently, CPAP has become the first line treatment for OSAS in adults and throat examination is not always performed systematically especially in cases with successful CPAP application. Hence, transoral examination is cheap, easy, quick and does not necessarily require specialized training. A widespread and easy screening examination tool of the oro‐pharyngeal area is the Mallampati score that evaluates the visibility of the uvula and soft palate when opening the mouth. The time‐benefit ratio of transoral screening examination is particularly high and not restricted to sleep apnea cases.^4^, ^5^

The present case suggests that sometimes patients may be equipped quickly with a CPAP even before or without having elaborated a careful search of origin for the OSAS. Inspection of the lateral pharyngeal walls is often and wrongly overlooked,^6^ and in the present case this would have saved one year of CPAP treatment. Although benign tumors of the parapharyngeal space are rare, they should not completely be forgotten. Especially when symptoms not classically found in OSAS are present, like the reported foreign body feeling in the present case, thorough ENT examination becomes mandatory.

We strongly emphasize routine transoral inspection before CPAP treatment and further underline the importance of careful physical ENT examination in OSAS workup.

## CONFLICT OF INTEREST

None of the authors declares any conflict of interests.

Patients consent for publication: Has been obtained.

## AUTHOR CONTRIBUTIONS

CAP and BNL: participated in the design and drafting of the manuscript. RG: operated and followed up the patient, obtained consent for publication, and helped in the conception of the manuscript.
